# ^13^C glucose labelling studies using 2D NMR are a useful tool for determining ex vivo whole organ metabolism during hypothermic machine perfusion of kidneys

**DOI:** 10.1186/s13737-016-0037-0

**Published:** 2016-08-05

**Authors:** Jay Nath, Tom Smith, Alex Hollis, Sam Ebbs, Sefa W. Canbilen, Daniel A. Tennant, Andrew R. Ready, Christian Ludwig

**Affiliations:** 1 Department of Renal Surgery, University Hospitals Birmingham, Birmingham, UK; 2 Institute of Metabolism and Systems Research (IMSR), College of Medical and Dental Sciences, University of Birmingham, Birmingham, UK

**Keywords:** Hypothermic machine perfusion, Kidney, Transplantation, Metabolism, NMR, Isotopic tracer study, ^13^C

## Abstract

**Background:**

The aim of this study is to determine the feasibility of using nuclear magnetic resonance (NMR) tracer studies (^13^C-enriched glucose) to detect ex vivo de novo metabolism in the perfusion fluid and cortical tissue of porcine kidneys during hypothermic machine perfusion (HMP).

**Methods:**

Porcine kidneys (*n* = 6) were subjected to 24 h of HMP using the Organ Recovery Systems LifePort Kidney perfusion device. Glucose, uniformly enriched with the stable isotope ^13^C ([U-^13^C] glucose), was incorporated into KPS-1-like perfusion fluid at a concentration of 10 mM. Analysis of perfusate was performed using both 1D ^1^H and 2D ^1^H,^13^C heteronuclear single quantum coherence (HSQC) NMR spectroscopy. The metabolic activity was then studied by quantifying the proportion of key metabolites containing ^13^C in both perfusate and tissue samples.

**Results:**

There was significant enrichment of ^13^C in a number of central metabolites present in both the perfusate and tissue extracts and was most pronounced for lactate and alanine. The total amount of enriched lactate (per sample) in perfusion fluid increased during HMP (31.1 ± 12.2 nmol at 6 h vs 93.4 ± 25.6 nmol at 24 h *p* < 0.01). The total amount of enriched alanine increased in a similar fashion (1.73 ± 0.89 nmol at 6 h vs 6.80 ± 2.56 nmol at 24 h *p* < 0.05). In addition, small amounts of enriched acetate and glutamic acid were evident in some samples.

**Conclusions:**

This study conclusively demonstrates that de novo metabolism occurs during HMP and highlights active metabolic pathways in this hypothermic, hypoxic environment. Whilst the majority of the ^13^C-enriched glucose is metabolised into glycolytic endpoint metabolites such as lactate, the presence of non-glycolytic pathway derivatives suggests that metabolism during HMP is more complex than previously thought. Isotopic labelled ex vivo organ perfusion studies using 2D NMR are feasible and informative.

## Background

Although there is good evidence to support the use of hypothermic machine perfusion (HMP) in clinical practice [1–3], there are surprisingly few reports detailing renal metabolism during this process. This is likely to reflect the widely held belief that metabolism is deleterious during organ preservation and should be therefore minimised by both hypothermia and keeping cold ischaemia times (CIT) as short as possible. The concept that metabolic support may have beneficial effects during HMP in addition to the mechanical effects is relatively recent [4, 5].

Using a one-dimensional (1D) ^1^H nuclear magnetic resonance (NMR) metabolomic approach, we have previously identified a panel of metabolites within the perfusion fluid during HMP that are predictive of post-transplant graft function [6]. We have also demonstrated that porcine and human metabolic profiles are similar, validating the pig as a valid metabolic model for kidney transplant studies [7].

However, the metabolic ‘snapshot’ provided by this conventional NMR metabolomic approach has limitations for the understanding of cellular metabolism in complex models such as ex vivo organ perfusion. Merely identifying and quantifying a metabolite is often insufficient in the determination of any reliable mechanistic information: even with serial measurements, such relationships are often unclear. For example, the detection of lactate within the perfusate of a machine-perfused kidney could be secondary to the metabolism or release of pre-existing intracellular substrate stores as well as de novo metabolism of substrates derived from the perfusion fluid. Thus, the appearance of a particular metabolite within the perfusion fluid does not confirm de novo metabolism. For this reason, we propose performing isotopic ^13^C-labelled glucose studies.

Incorporation of stable isotopes such as ^13^C into common compounds enables metabolic tracer studies within a biological system. This is not a new concept, with initial studies reported over 40 years ago [8]. Introduction of labelled metabolic precursors can highlight specific pathways within the metabolic network. For example, after addition of [U-^13^C] glucose to the fluid of a kidney during HMP, detection of uniformly labelled ^13^C lactate provides unequivocal evidence of glycolytic pathway activity.

NMR spectroscopy is a powerful tool to analyse complex ^13^C isotopomer/isotopologue distributions in metabolites derived from labelled tracer molecules. There are various spectroscopic methods available, of which the simplest is a 1D ^13^C approach. 1D NMR organ perfusion studies have been reported in ex vivo heart and lung animal models using perfusate labelled with ^13^C glucose and pyruvate [9–12]. This demonstrates the feasibility of using ^13^C-labelled metabolic precursors to determine de novo metabolism in a whole organ perfusion model. 2D ^1^H,^13^C HSQC NMR is an alternative to 1D ^13^C NMR, benefitting from higher sensitivity and increased spectral dispersion. Whilst previous authors have demonstrated the utility of a human 2D NMR approach using ^13^C-labelled glucose [13], to our knowledge there are no reports of this technique in the field of transplantation.

The aim of this study is to assess whether metabolic pathway activity within a machine-perfused porcine kidney can be determined using ^13^C-labelled glucose tracer experiments and 2D NMR.

## Methods

### Pig studies

**Fig. 1:**
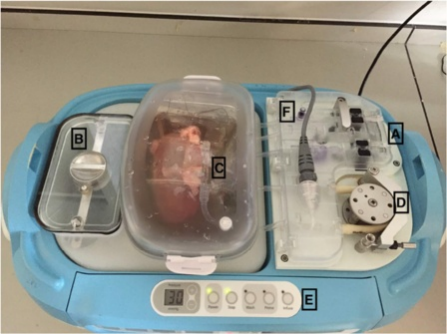
LifePort Kidney Transporter 1.0 (Organ Recovery Systems) containing a porcine kidney. **a** Bubble chamber. **b** Reservoir for ice. **c** Aortic patch connector for perfusion. **d** Peristaltic pump. **e** Controls (pressure, prime, wash, infuse). **f** Sampling port

Experiments were performed on 22–26-week-old ‘bacon weight’ pigs, weighing 80–85 kg (*n* = 6) as described elsewhere [7]. All experiments were performed following the principles of laboratory animal care according to NIH standards. To replicate human conditions, kidneys were initially cold flushed (4 °C) with 1 l Soltran solution under aseptic conditions at a pressure of 150 mmHg, followed by 2 h of cold storage. Organs were then perfused with 1 l of a modified version of KPS-1 solution containing 10 mM of [U-^13^C]-labelled glucose and 30 g of PEG 35 kDa as an impermeant in place of hydroxyethyl starch. The LifePort Kidney Transporter 1.0 (Organ Recovery Systems) was used for perfusion at a pressure of 30 mmHg (Fig. 1).

Kidneys were perfused for 24 h with perfusate sampled at times 6, 12, 18 and 24 h from each kidney via the designated LifePort sampling port and stored at −20 °C. The samples were thawed at room temperature, prepared and processed as described below. Kidney cortex tissue samples were also taken at the study endpoint and flash frozen prior to homogenization.

### 2D NMR spectroscopy

**Fig. 2:**
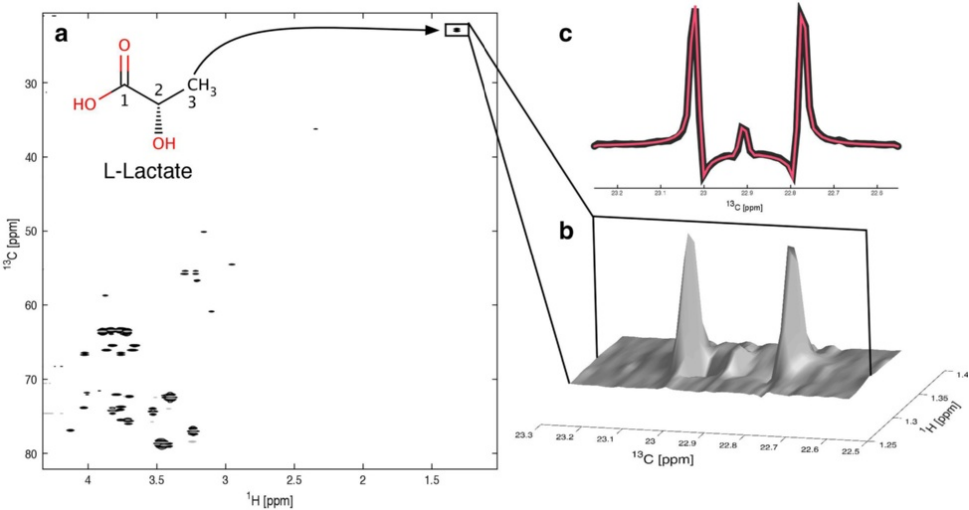
A 2D HSQC spectrum of extracted pig kidney cortex is shown in **a**. A 3D plot of the methyl group (carbon 3) signal of lactate is displayed in **b.** **c** A cross section through the middle of the methyl group signal. The recorded spectrum is plotted in *black*, whereas a quantum-mechanical simulation of the same multiplet is shown in *red*. The plot demonstrates that the simulation allows for a fully quantitative analysis of the NMR multiplets, which forms the base for a subsequent isotopomer/isotopologue analysis

**Fig. 3:**
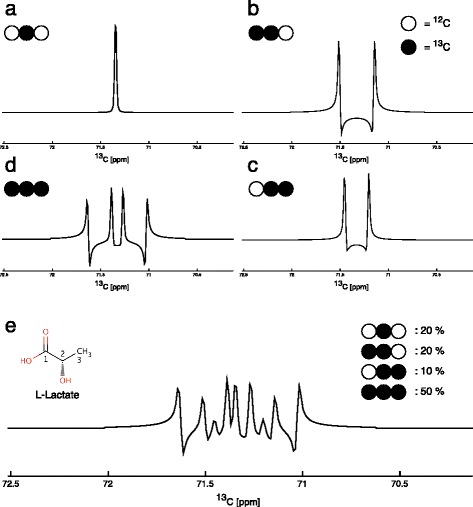
Possible multiplet components for the central carbon (carbon 2) of lactate. Clockwise (**a–d**), the four possible multiplet components are shown. The spectrum shown in **a** contains ^13^C in position 2 only, whereas positions 1 and 3 carry a ^12^C nucleus. In both **b** and **c**, one of the adjacent carbon nuclei carries an additional ^13^C nucleus in the same molecule. Therefore, the NMR signal is split into two resonance lines (doublet). Because of the different chemistry of carbons 1 and 3, the amount of splitting depends on which neighbour carries the ^13^C nucleus. This is why splittings can be used to uniquely assign which neighbouring carbon is labelled even though it may be impossible to directly observe that carbon, such as carbon 1, in a HSQC spectrum. The multiplet for a fully labelled lactate molecule is plotted in **d**. Because both neighbouring carbons are ^13^C, the signal is now split into four peaks (doublet of doublets). Under normal circumstances, multiplets will contain a mixture of those patterns. Such a mixture is shown in **e**. Despite the rather complex appearance of this signal, it can be fully quantified by a quantum-mechanical signal deconvolution

2D HSQC NMR experiments were performed in this study. This type of 2D NMR exploits the spin properties of both protons (^1^H) and isotopic carbon (^13^C) such that only ^13^C nuclei with an attached ^1^H produce a signal on the resulting spectrum (Fig. 2). Whilst the pulse sequence used here actively decouples ^1^H and ^13^C, inter-proton and inter-carbon couplings are still active. However, the only coupling resolved in the resulting NMR spectra is the ^13^C–^13^C coupling. Thus the spectral peak of a ^13^C nuclei is altered if there is an adjacent ^13^C nuclei, in that the signal is split and two peaks exist, splitting into four if flanked by a ^13^C either side (Fig. 3). In these experiments, [U-^13^C] glucose was used as a tracer (i.e. ^13^C nuclei in all six carbon positions of the glucose molecule) therefore forming lactate with all three carbons in the isotopic ^13^C version produced through glycolysis. As the natural abundance of ^13^C is known (1.1 %) and the likelihood of adjacent ^13^C nuclei occurring naturally very low (1.1 %^2^ = 0.0121 %), it is possible to determine the relative concentration of isotopic carbon molecules compared to the natural abundance levels by performing quantum-mechanical simulations of the different components of an experimental HSQC multiplet (Fig. 3). Absolute concentrations of metabolites can be determined using 1D ^1^H NMR, and therefore, absolute concentrations of labelled metabolites can be calculated using a combination of these two NMR approaches.

### Sample preparation

#### Perfusates

Perfusate samples were extracted using a chloroform/methanol extraction. Briefly, 1.5 ml of perfusate was combined with 1.5 ml of chilled methanol (at −80 °C) and 1.5 ml chloroform (i.e. a 1:1:1 ratio). This was mixed vigorously for 10 min and then cooled on wet ice for 10 min. The polar and non-polar phases were separated by centrifugation (1300*g* for 15 min at 4 °C) as described elsewhere [14, 15] and 2 ml of the polar fraction aspirated and dried at 30 °C overnight. The dried samples were resuspended in 480 μl NMR phosphate buffer containing 0.1 M phosphate buffer (pH 7.0), 0.5 μM DSS (4,4-dimethyl-4-silapentane-1-sulfonic acid), 2 mM imidazole, and 48 μl D_2_0 (deuterium oxide), and vortexed until the dried pellet had completely dissolved. Following this, 35 μl of the sample was added to a 1.7-mm NMR tube, sonicated for 10 min to dissolve any remaining micro particles and then centrifuged to remove air bubbles. The sample was stored at 4 °C until processed.

#### Tissues

Immediately after storage, the kidneys were laterally bisected and sections of cortex were excised and snap frozen in liquid nitrogen. Sections of cortex were then pulverised to a fine powder using a manual cryogrinder. After this, 0.5 g of the powdered sample was added to a 7-ml Precellys homogenisation tube containing 5.1 ml of chilled methanol (−80 °C) to quench ongoing metabolism. The sample was homogenised using the Precellys 24 Dual homogeniser set at the lowest mode (5000 rpm for 15 s) until fully homogenised (this was standardised to eight courses of the lowest setting with cooling in dry ice between runs to prevent overheating). Once homogenised, the samples were mixed with 4.65 ml ultrapure water accounting for the estimated 79 % water (around 395 μl) present in the tissue already [16] and 5.1 ml chloroform (i.e. a 1:1:1 ratio as with the perfusate samples).

Metabolite extraction and resuspension in NMR buffer was performed as in the perfusate sample preparation, with the exception that 4.5 ml of the top polar layer was aspirated and dried before resuspension in 60 μl of NMR phosphate buffer and 35μl of this was added to a 1.7mm NMR tube as before.

### NMR spectroscopy

**Fig. 4:**
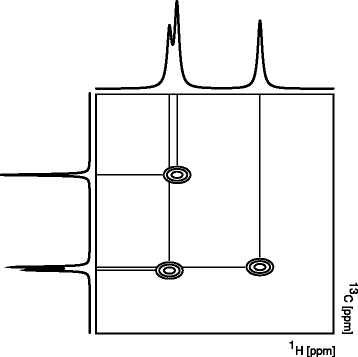
Schematic representation of 2D HSQC NMR spectrum. Chemical shift for ^1^H depicted on the *x*-axis and ^13^C on the *y*-axis. Whilst in both the projected 1D ^1^H and the 1D ^13^C spectrum display signal overlap, all signals are well resolved in the 2D spectrum

Both 1D ^1^H NMR and 2D ^1^H,^13^C HSQC NMR spectra were acquired on a 600-MHz Bruker Avance III NMR spectrometer equipped with a TCI 1.7-mm z-PFG cryogenic probe at 300 K. NMR acquisition time was circa 5 h per sample (Fig. 4). The spectral widths for all spectra were set to either 7812.5 Hz (^1^H) or 24,155 Hz (^13^C). For the 1D ^1^H NMR spectra, 16,384 complex data points were acquired. For the ^1^H dimension of 2D ^1^H,^13^C HSQC NMR spectra, 512 complex data points were acquired. Out of 8192 complex data points (2458), 30 % were sampled for the ^13^C dimension using an exponentially weighted non-uniform sampling scheme. Using a 4-s interscan relaxation delay, 128 transients were recorded for the 1D NMR spectra. Two transients per increment were recorded for the 2D ^1^H,^13^C HSQC NMR spectra. The interscan relaxation delay was set to 1.5 s. Each sample was automatically tuned, matched and then shimmed (1D TopShim) to a DSS line width of <1 Hz prior to acquisition of the first spectrum. 1D ^1^H NMR spectra were processed using the MATLAB-based MetaboLab software [17]. All 1D data sets were zero-filled to 131,072 data points prior to Fourier transformation. The chemical shift was calibrated by referencing the DSS signal to 0 ppm. 1D spectra were manually phase corrected before correcting the spectral baseline using a spline function [17]. 1D ^1^H NMR spectra were then exported into Bruker format for metabolite identification and concentration determination using Chenomx 8.1 (ChenomxINC). 2D ^1^H,^13^C HSQC NMR spectra were reconstructed with compressed sensing using the MDDNMR and NMRpipe software [18–20]. The spectra were zero-filled to 1024 (^1^H) times 16,384 (^13^C) real data points. HSQC spectra were then analysed using MetaboLab, which uses the pyGamma software for multiplet simulations [21]. The methyl group of lactate was used to calibrate the chemical shift based on its assignment in the human metabolome database [22].

### Statistical analysis

Data were analysed using GraphPad Prism v6 (GraphPad Software, San Diego, USA). Data were reported as mean ± SD, unless stated otherwise. Statistical significance was tested using two-tailed Student’s *t* test. A *p* value of <0.05 was considered significant.

## Results

An understanding of the active metabolic pathways during HMP could potentially facilitate the development of a more nutritionally supportive perfusion fluid enabling the metabolic optimisation of these valuable organs prior to transplantation.

In this donation after cardiac death (DCD) large animal model of kidney transplant preservation, enrichment of ^13^C in a number of metabolites was observed in both perfusate solution and kidney cortex tissue. This confirms that de novo metabolism occurs during HMP and that the proposed NMR methodology is useful to demonstrate this. Even after 6 h of perfusion, glycolytic activity was evident with enriched [U-^13^C] lactate, enriched [U-^13^C] alanine and enriched [U-^13^C] acetate detected in the perfusion fluid.

The proportion of [U-^13^C] enriched lactate (as a fraction of the total lactate present) increased over the study period (3.25 % ± 1.02 after 6 h and 7.74 % ± 1.57 after 24 h *p* < 0.001). In addition, the total concentration of lactate present (enriched and non-enriched) increased (1.51 ± 0.20 mM at 6 h and 2.04 ± 0.57 mM at 24 h *p* < 0.05). Given that the total amount of enriched lactate present at a given time point is the product of the relative proportion (e.g. 3.25 %) and total concentration (e.g. 1.51 mM), it is self-evident that as both of these are increasing; the amount of enriched lactate being produced is also increasing (total amount of labelled [U-^13^C] lactate (per sample) 31.1 ± 12.2 nmol at 6 h vs 93.4 ± 25.6 nmol at 24 h *p* < 0.01).

**Fig. 5:**
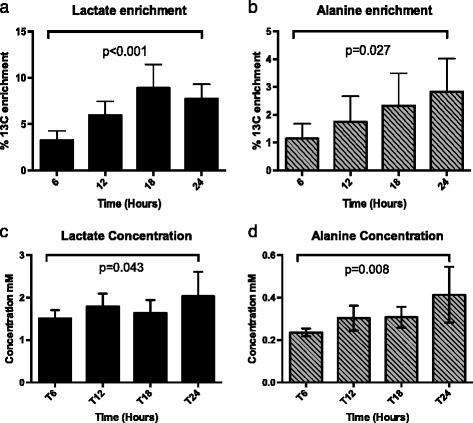
Changes over time for lactate and alanine in the perfusion fluid of HMP kidneys. **a**, **b** The % enrichment present and **c**, **d** the total concentration of these metabolites. In all cases, there was a significant increase both in ^13^C enrichment and in concentrations between the 6- and 24-h samples

There is a similar pattern observed with alanine with the enriched proportion increasing (1.25 % ± 0.44 at 6 h and 2.83 % ± 1.19 at 24 h *p* < 0.05) and the total concentration increasing by a similar proportion (0.24 mM ± 0.018 at 6 h and 0.41 mM ± 0.13 at 24 h *p* < 0.01) (total [U-^13^C] alanine per sample (1.73 ± 0.89 nmol at 6 h vs 6.80 ± 2.56 nmol at 24 h *p* < 0.05)) (Fig. 5).

Whilst enriched acetate was present at all time points in the perfusion fluid (circa 1.25 %), there was no change in concentration over time. Similarly, the total concentration of acetate did not change over time, suggesting that either the production of acetate was limited or being converted to other enriched substrates.

The same enriched metabolites (lactate, alanine and acetate) were also present in the extracted kidney cortex samples at all time points. In addition, glutamate with ^13^C enrichment in the 4 and 5 carbon position was present in several samples in small amounts (<0.5 % of total present). The presence of this labelling pattern in glutamate indicates mitochondrial tricarboxylic acid (TCA) cycle activity (via pyruvate dehydrogenase and complex 1).

## Discussion

This paper provides further evidence that a significant amount of metabolism occurs during HMP and that NMR methods are useful for identifying these [6, 7, 23]. However, unlike ^1^H NMR reports, this paper provides unequivocal evidence that de novo metabolism occurs in these organs during HMP with the glucose present in the perfusate used as a substrate. To our knowledge this is the first report to demonstrate such de novo metabolism in a whole organ ex vivo kidney model using NMR tracer methods.

The labelled tracer study techniques described herein highlight active metabolic pathways during the machine perfusion period. This could be particularly useful as clinicians seek to alter perfusion characteristics in order to optimise kidney graft function prior to transplant. The metabolic effects of such perfusion modification (e.g. temperature, oxygenation, oxygen carriers) can be elegantly demonstrated using ^13^C NMR, with analysis of perfusion fluid permitting non-invasive metabolic monitoring.

The appearance of uniformly labelled lactate and alanine in the perfusate and tissue samples demonstrate that de novo glycolysis is occurring in this non-physiological environment and that alternative metabolic pathways such as the pentose phosphate pathway do not appear to be active. Although cellular metabolism is reported to only be about 5–8 % at temperatures below 4 °C [24] with similar oxygen requirements [25], hypothermia does not cause uniform deceleration of all metabolic pathways [4] and the metabolic effects of hypothermia within different organs is not uniform [26]. There is some evidence that the energy substrate utilised to support metabolism in a hypothermic canine ex vivo model is predominantly glucose entering glycolysis [27], highlighting the importance of this pathway during machine preservation of organs. Interestingly, other studies demonstrate an increase in perfusate metabolites such as glutamate during perfusion. The suggested mechanism was the reduction (and deamination) of glutamine to glutamate by glutaminase. The authors concluded that the deamination of glutamate occurs to form alpha-ketoglutarate, an important TCA cycle intermediate [28].

The presence of labelled acetate within the perfusate is interesting. Whilst acetate can be formed from multiple pathways, the early appearance of the labelled substrate in these experiments may indicate acetyl-coA hydrolase activity [29].

The labelled metabolites in this study were detectable in the extracellular perfusion fluid within 6 h and demonstrates that cellular uptake of glucose, glycolysis and transport/efflux of resulting metabolites occurs within this time period. Although the proportion of de novo metabolites is modest, it does occur independently of any glucose uptake stimulators such as insulin.

There has been great interest in the role of oxygenation and oxygen carriers during kidney preservation, with proponents suggesting that oxygenation replenishes cellular ATP levels [30] and detractors highlighting the perils of uncontrolled oxygenation exacerbating the reactive oxygen species (ROS) generation [31] and the ischaemia reperfusion phenomenon [32]. This study demonstrates that even in the absence of supplemental oxygen, small amounts of glucose-derived glutamic acid were observed in several tissue samples indicative of aerobic TCA cycle activity. Although this is surprising, it must be remembered that HMP is not an anoxic environment and oxygen solubility increases at lower temperatures and increased salinity and the preservation solution is further saturated by the physical fluid agitation that occurs during perfusate cycling. Whilst other metabolites indicative of TCA activity were commonly identified using NMR such as citrate, succinate and fumarate (by 2D and/or 1D), they were not labelled to indicate de novo formation.

One of the benefits of the 2D NMR methodology used in this paper is that the sensitivity (per unit time) is increased compared with 1D ^13^C NMR. However, ^13^C NMR is inherently less sensitive than ^1^H NMR, and therefore, it is possible that labelled metabolites present in very small quantities would not be identified using this method. Furthermore, our 2D database of splitting patterns and chemical shift parameters for different metabolites is evolving and other labelled metabolites may be identified once these have been further clarified. Whilst we feel the 2D NMR methods in this paper relay unparalleled amounts of metabolic information, they are both time consuming and resource intensive and would not be suitable for real-time analysis to inform clinical decisions, as proposed for 1D ^1^H NMR studies [23].

The experimental conditions in these experiments were standardised as much as possible to reduce inter-experimental variability and designed to replicate clinical conditions. Whilst previous studies have repeatedly demonstrated the viability of porcine kidneys after similar periods of HMP [23, 33], this study does not seek to corroborate this and as such we are unable to correlate de novo metabolism with functional outcome. Given the findings from earlier studies [6], we hypothesise that glycolytic activity during perfusion may correlate with post-transplant function and propose a human observational study to clarify this, which is possible due to the non-radioactive nature of the ^13^C isotope used in this study.

## Conclusions

We conclude that the described ^13^C labelling study convincingly demonstrates de novo metabolism during kidney storage by HMP, and this can be used to highlight active metabolic pathways in this hypothermic, hypoxic environment.

We postulate that although most of the supplied ^13^C-enriched glucose is converted to endpoint metabolites associated with glycolytic metabolism (i.e. lactate), the presence of labelled non-glycolytic pathway derivatives suggests that kidney metabolism during HMP storage is more complex than previously thought.

Isotopic labelled ex vivo organ perfusion studies using 2D NMR are therefore informative and feasible. Metabolic manipulation is a potential target for therapeutic intervention during the preservation period, and accurate understanding of active metabolic pathways could potentially facilitate studies to optimise this, potentially improving the function of the kidneys post-transplant.

## Abbreviations

CIT, cold ischaemia time; HMP, hypothermic machine perfusion; HSQC, heteronuclear single quantum coherence; NIH, National Institute of Health; PEG, polyethylene glycol
